# Chromatin versus pathogens: the function of epigenetics in plant immunity

**DOI:** 10.3389/fpls.2015.00675

**Published:** 2015-09-02

**Authors:** Bo Ding, Guo-Liang Wang

**Affiliations:** ^1^State Key Laboratory of Plant Diseases and Insect Pests, Institute of Plant Protection, Chinese Academy of Agricultural SciencesBeijing, China; ^2^Department of Plant Pathology, The Ohio State University, ColumbusOH, USA

**Keywords:** Chromatin, histone modification, chromatin remodeling, plant immunity

## Abstract

To defend against pathogens, plants have developed a sophisticated innate immunity that includes effector recognition, signal transduction, and rapid defense responses. Recent evidence has demonstrated that plants utilize the epigenetic control of gene expression to fine-tune their defense when challenged by pathogens. In this review, we highlight the current understanding of the molecular mechanisms of histone modifications (i.e., methylation, acetylation, and ubiquitination) and chromatin remodeling that contribute to plant immunity against pathogens. Functions of key histone-modifying and chromatin remodeling enzymes are discussed.

## Introduction

Throughout their life cycles, plants are exposed to abiotic stresses, including temperature fluctuation and nutrition deficiency, and biotic threats, including attack by herbivores and microbial pathogens. With respect to microbial pathogens, plants are unlike animals in that they lack an adaptive immune system that produces antibodies and also lack mobile circulatory cells that detect and prevent or reduce infection. Instead, plants mainly rely on an innate immunity system to resist microbial attack. In plants, the salicylic acid (SA), jasmonic acid (JA), and ethylene (ET) signaling pathways play pivotal roles in defending against biotrophic and necrotrophic pathogens (Pieterse et al., 2009). After detecting a pathogen, the plant activates a cascade of defense responses to establish local and systemic acquired resistance (SAR; Durrant and Dong, 2004).

Transcription of defense genes is tightly regulated by many transcription factors (TFs) that fine-tune the defense response (Thilmony et al., 2006). This requires that plants rapidly and precisely re-program gene expression. In particular, activation of an appropriate stress signaling pathway following pathogen detection is integrated in the plant cell nucleus through a set of regulatory cascades that prioritize defense over growth-related cellular functions (Moore et al., 2011). Research over the last decade has revealed that this transcriptional re-programming and regulation of defense-related genes often involves chromatin modifications and remodeling in *Arabidopsis* (Alvarez et al., 2010). In this review, we summarize and discuss the roles of chromatin modifications and remodeling in plant defense.

## Plant Innate Immunity

Plant innate immunity is triggered by pattern recognition receptors (PRRs) located on the external cell surface. PRRs can recognize specific pathogen-/microbe-associated molecular patterns (PAMPs/MAMPs), including cell wall components, short peptides, and lipopolysaccharides derived from the pathogen, leading to PAMP/MAMP-triggered immunity (PTI/MTI), which is the first layer of defense (Boller and Felix, 2009). Several early defense responses, including the generation of reactive oxygen species (ROS), calcium flux, plant cell wall modification, and the activation of a cascade of mitogen-activated protein kinases (MAPKs), are triggered during PTI. To overcome PTI, pathogens may deliver effector proteins into host cells, resulting in effector-triggered susceptibility (ETS). An additional level of resistance associated with vigorous defense induction may occur when specific intracellular receptors/sensors called resistance (R) gene products to recognize such race-specific avirulence (avr) effectors, thereby activating effector-triggered immunity (ETI; Jones and Dangl, 2006). ETI usually triggers a localized cell death at the infection site, in a process known as the hypersensitive response (HR), which along with antimicrobial effects may restrict most pathogen growth (Caplan et al., 2008). In addition to these PTI and ETI responses after initial local infection, the uninfected portions of the plant usually develop SAR, providing resistance in distal plant tissues against subsequent pathogen challenges (Durrant and Dong, 2004; Mishina and Zeier, 2007).

## Chromatin Modification in Plant Innate Immunity

### Chromatin Structure and Modifications

The basic, repeated unit of chromatin is the nucleosome that contains 147 base pairs (bp) of DNA wrapped around a histone octamer, which in turn consists of two copies of the following core histones: H2A, H2B, H3, and H4 (Luger et al., 1997). The linker histone, H1, associates with DNA between two nucleosomes and participates in higher order chromatin structure formation and remodeling. Extending from the globular nucleosome core, the histone tails may harbor diverse post-translational modifications (PTMs), i.e., acetylation, methylation, phosphorylation, ubiquitination, sumoylation, carbonylation, and glycosylation. PTMs can directly affect chromatin structure or can recruit specific “readers or effectors,” thereby regulating gene expression mainly by altering nucleosome stability and positioning, which affect the accessibility for regulatory proteins or protein complexes involved in transcription, DNA replication, and repair (Kouzarides, 2007). In general, histone acetylation by histone acetyltransferases (HATs) is associated with transcriptional activation, while histone deacetylation by histone deacetylases (HDACs) is associated with transcriptional suppression (Eberharter and Becker, 2002). Depending on the context of targets, histone methylation and/or ubiquitination can either be an active or repressive marker for transcription. Generally, tri-methylations of H3K4 and H3K36 (H3K4me3 and H3K36me3) and mono-ubiquitination of H2B (H2Bub) are enriched at actively expressed genes (Xu et al., 2008; Zhang et al., 2009), H3K27me3 is associated with repressed genes, while H3K9me2 and H4K20me1 are enriched at constitutive heterochromatin and silenced transposons (Zhang et al., 2007a,b; Bernatavichute et al., 2008). In addition to histone modification, ATP-dependent chromatin-remodeling enzymes use the energy of ATP hydrolysis to remodel chromatin structure by modifying the interaction between DNA and histone to relocate or dissociate nucleosomes, move histone octamers, and catalyze the incorporation of specific histone variants. ATP-dependent chromatin-remodeling enzymes thus play crucial roles in nucleosome assembly/disassembly and allow the transcriptional machinery to access the DNA (Smith and Peterson, 2005; Clapier and Cairns, 2009).

Many studies have documented that histone modifications and ATP-dependent chromatin remodeling result in rapid, reversible, or trans-generational changes in gene expression associated with various developmental processes, such as flowering time control, cell fate determination and maintenance, and seed development. These mechanisms, however, have only recently attracted attention as potential transcriptional regulators in plant innate immunity (**Table 1**).

**Table 1:** Histone-modifying enzymes and chromatin-remodelling factors involved in plant responses to pathogens.

Modification category	Sub-category	Name	Gene locus	Mutant phenotype and biological role	Reference
Histone acetylation	Histone deacetylase (HDAC)	*HDA19/AtHD1*	At4G38130	Increases sensitivity to *Alternaria brassicicola* and *Pst* DC3000; down-regulates ET/JA pathway genes (PDF1.2, VSP2, and ERF1), and enhances basal expression of SA-responsive genes (PR1, PR4, and PR5)	Zhou et al. (2005), Kim et al. (2008), Choi et al. (2012)
		*HDA6/Axe1*	At5G63110	Down-regulates expression of ET/JA pathway genes (PDF1.2, VSP2 ERF1)	Zhou et al. (2005)
		*AtSRT2*	At5G09230	Increases resistance to *Pst* DC3000; down-regulates expression of SA-biosynthesis genes (PAD4, EDS5, and SID2)	Wang et al. (2010)
		*HDT701*	Os5G51830	Increases resistance to rice blast in RNAi plants; up-regulates mitogen-activated protein kinases (MAPK6), WRKY53	Ding et al. (2012)
	Histone acetylase	*HAC1*	At1G79000	Mutants deficient in priming the of PTI	Singh et al. (2014a)
Histone methylation	Histone methytransferase	*ATX1/SDG27*	At2G31650	Down-regulates expression of SA-pathway genes (WRKY70 and PR1); up-regulates expression of ET/JA pathway genes (PDF1.2, VSP2)	Alvarez-Venegas et al. (2007)
		*SDG8/ASHH2/EFS/LAZ2*	At1G77300	Increases sensitivity to *Botrytis cinerea*; down-regulates expression of ET/JA pathway genes; increases sensitivity to *PST* DC3000, down-regulates the basal expression of R genes (LAZ5 and RPM1) and SA-inducible genes (WRKY70 and PR1)	Berr et al. (2010), Palma et al. (2010), De-La-Pena et al. (2012)
		*ASHR1*	At2G17900	Increases sensitivity to *Pst* DC3000, down-regulates the expression of SA-inducible genes (WRKY70 and PR1)	De-La-Pena et al. (2012)
	Histone demethylase	*FLD/RSI1*	At3G10390	Decreases resistance after systemic acquired resistance (SAR) induction, down-regulates expression of SAR-inducible WARY6 and WRKY29	Singh et al. (2013, 2014b)
		*JMJ705*	Os1G67970	Increases sensitivity to *Xoo*, down-regulates the basal and MeJA-inducible defense genes	Li et al. (2013)
Histone ubiqutination	H2B ubquitation-ligase	*HUB1*	At2G44950	Increases sensitivity to *B. cinerea* and *A. brassicicola*, does not alter expression of PDF1.2; decreases resistance to *Pst* DC3000 in *snc1* and *bon1* background, down-regulates the expression of the R gene SNC1	Dhawan et al. (2009), Zou et al. (2014)
Chromatin remodeling factors	SWI2-like group	*DDM1*	At5G66750	Increases resistance to Pst DC3000 in *mos1/snc1* background, up-regulates the expression of R gene SNC1	Li et al. (2010)
	SWR1-like group	*PIE1/CHR13*	At3G12810	Enhances resistance to *Pst* DC3000, up-regulates the expression of SA-pathway genes	March-Diaz et al. (2008)
	SNF2-like group	*SYD/CHR3*	At2G28290	Increases sensitivity to *B. cinerea*, down-regulates expression of ET/JA pathway genes (PDF1.2, VSP2, and Myc2)	Walley et al. (2008)

### Histone Acetylation

Histone lysine acetylation is regulated by the antagonistic interactions between HATs and HDACs. Plant HDACs can be divided into four major groups or families. In addition to a plant-specific type-II HDAC (HD2) family, three other major families are designated as reduced potassium dependency 3 (RPD3), HDA1, and silence information regulator 2 (SIR2); this grouping is based on homology to yeast counterparts. Among these groups, HDA19 from *Arabidopsis* has been well-studied with regard to its roles in plant defense against pathogen attack. HDA19, which belongs to the RPD3 subfamily, was initially reported to be involved in the ET/JA signaling pathways of defense responses based on two lines of evidence. First, the expression of *HDA19* is induced by wounding, by challenge with the pathogen *Alternaria brassicicola*, and by treatment with the plant hormone JA. Second, the knock-down mutant of *HDA19* exhibits decreased transcription of several ET/JA pathway genes (*ERF1, CHI-B*, and *BGL*) and increased susceptibility to fungal pathogens, while overexpression results in the opposite disease phenotypes (Zhou et al., 2005). Similarly, HDA6, another *Arabidopsis* RPD3-type HDAC, is induced by treatments with JA and the ET precursor ACC, whereas the expression of other members of *Arabidopsis* RPD3-type HDACs is not inducible by these hormones (Zhou et al., 2005). In addition, HDA6 interacts with an F-box protein, coronatine insensitive 1 (COI1), which mediates JA signaling (Devoto et al., 2002). The expression of the JA-responsive genes, i.e., *PDF1.2, VSP2, JIN1*, and *ERF1*, is down-regulated in *axe1-5* (HDA6 loss-of-function mutant) and HDA6-RNAi plants (Wu et al., 2008), suggesting redundant roles of HDA6 and HDA19 in plant defense against infection by necrotrophic pathogens. In addition to its role in the JA/ET defense pathway, HDA19 positively regulates SA-mediated basal defense and the expression of pathogenesis-related gene 1 (PR1) by physically interacting with WRKY38 and WRKY62 and inhibiting their transcriptional-activator activities (Kim et al., 2008). On the other hand, the basal expression of the SA-induced *PR1* and *PR5* is upregulated in the *hda19* mutant when it is not challenged by pathogens, reflecting the negative role of HDA19 in defense responses. *PR1* and *PR2* are well-defined markers for SA-mediated basal and R gene-mediated defense against biotrophic pathogens (Ward et al., 1991; Rairdan and Delaney, 2002; van Loon et al., 2006). Several studies have shown that the SA-induced activation of *PR1* is tightly correlated with an increase in the level of acetylated histones at the *PR1* locus in *Arabidopsis* (Mosher et al., 2006; Koornneef et al., 2008) and tobacco (Butterbrodt et al., 2006). Additionally, HDA19 associates directly with the promoters of *PR1* and *PR2* and deacetylates histones at *PR1* and *PR2* locus. Thus, HDA19 forms a repressive chromatin environment (low histone acetylation level) under unchallenged conditions that ensures a low basal expression of defense genes as well as the proper induction of *PR* genes without harmful overstimulation during defense responses to pathogen attacks (Choi et al., 2012).

The HDAC proteins in the Sir2 family are NAD+-dependent HDACs that play diverse roles in a variety of physiological processes, including chromatin silencing, DNA repair, the cell cycle, and apoptosis and aging in yeast and mammalian systems (Eberharter and Becker, 2002; Yamamoto et al., 2007; Etchegaray et al., 2013). Both *Arabidopsis* and rice genomes contain two Sir2 family genes (Pandey et al., 2002). Knockdown of *OsSRT1* by RNAi in rice plants enhances histone H3K9 acetylation on the promoters of HR-related genes, which leads to hydrogen peroxide accumulation, DNA fragmentation, and cell death, suggesting a negative role of *OsSRT1* in defense (Huang et al., 2007). Highly divergent in sequence from *OsSRT1, AtSRT2* is down-regulated by *Pseudomonas syringae* pv. *tomato DC3000* (*Pst DC3000*) infection and negatively regulates the plant basal defense and *PR1* expression, possibly by suppressing pathogen-induced expression of *PAD4, EDS5*, and *SID2* and thereby regulating SA synthesis (Wang et al., 2010).

In addition to local resistance, SAR is also related to priming for stronger activation of various defense responses that are induced following an attack by microbial pathogens (van Hulten et al., 2006). Priming of innate immunity is correlated with chromatin modification of the promoter region of WRKY TF genes (Jaskiewicz et al., 2011) and SA- and PTI-responsive genes (Luna et al., 2012; Po-Wen et al., 2013). Researchers recently showed that repetitive abiotic stress causes the priming of PTI in *Arabidopsis*, leading to enhanced resistance to bacterial pathogens. This elevated defense after repeated exposure to environmental stress is compromised in the *hac1* mutant, establishing a link between open chromatin configuration such as HAC1-dependent histone acetylation and primed *Arabidopsis* innate immunity and bacterial resistance (Singh et al., 2014a).

### Histone Methylation

The *Arabidopsis* genome encodes 37 putative SET-domain group proteins, some of which have been experimentally demonstrated to harbor histone methyltransferase (HMT) activity (Thorstensen et al., 2011). For the removal of methyl residues from the methylated histones, the lysine-specific demethylase 1 (LSD1)-like proteins and Jomonji C-domain (JmjC) proteins are effective in histone demethylation in plants (Chen et al., 2011). Dynamic histone methylation and de-methylation are involved in many cellular processes such as gene imprinting and DNA methylation (Kohler et al., 2012), and in developmental events such as vernalization (Kim and Sung, 2014). Recent findings indicate that histone methylation contributes to plant immunity against both necrotrophic and biotrophic pathogens by affecting the expression of specific NBS-LRR proteins, WRKY family TFs, as well as TFs involved in defense signaling pathways.

The first study of immune responses involving histone methylation concerned *Arabidopsis* trithrox 1 (ATX1), also known as SDG27. Loss of *ATX1* function affects the transcription of a subset of pathogen- and disease resistance-associated genes, including those encoding members of the TIR-NBS-LRR classes of disease resistance proteins, lectins, and heat shock proteins, as well as several WRKY family TFs (Alvarez-Venegas et al., 2006). Further findings revealed that ATX1 directly controls H3K4me3 levels at the promoter of *WRKY70* and also controls the expression of *WRKY70*, a positive regulator of SA-mediated defense signaling against bacterial pathogens (Alvarez-Venegas et al., 2007; Saleh et al., 2008). *Arabidopsis* trithorax-related 7 (Atxr7), another histone H3K4 methyltransferase in the trithrox1 group, physically associates with the modifier of *snc1* 9 (MOS9), which is a plant-specific protein with unknown function discovered in a forward genetic screening of the *snc1* mutant. Together with MOS9, Atxr7 is required for both maintaining the H3K4me3 levels at the promoter of the NBS-LRR genes *Snc1* and *Rpp4* and expression of these R genes residing in the RPP4 cluster (Xia et al., 2013).

In *Arabidopsis*, another important active signature of histone modification, H3K36 tri-methylation, is catalyzed by the SET domain group 8 (SDG8, also known as EFS, LAZ2, and Ashh2; Xu et al., 2008). SDG8 is a homolog of SET2 in yeast and ASH1 in *Drosophila*. Mutation in *SDG8* causes pleiotropic developmental phenotypes such as early flowering time, reduced organ size, and enhanced branch shooting (Zhao et al., 2005; Dong et al., 2008; Cazzonelli et al., 2009). A recent study revealed that *sdg8* mutant plants have reduced resistance to the necrotrophic fungal pathogens *A. brassicicola* and *Botrytis cinerea*, indicating that SDG8 plays a crucial role in plant defense through H3K36me3-mediated activation of a subset of genes (including *ERF1, PDF1.2a*, and *VSP2*) in the JA/ET signaling pathways (Berr et al., 2010). Another study showed that SDG8 is required for both basal and R-protein-mediated resistance and that SDG8 maintains the *LAZ5* locus in a transcriptionally active state by modifying its H3K36me3 level. LAZ5 is a member of an immune receptor class involved in the detection of specific pathogens and subsequent cell death (Palma et al., 2010). In a comparative analysis of three *Arabidopsis* ASH1 family mutants, loss of function of *ASHH2* and *ASHR1* resulted in more rapid HRs to both a non-pathogenic strain (*hrpA-*) and a pathogenic strain (*DC3000*) of *P. syringae*. In contrast, the *ashr3* mutant is more resistant to the infection than the *ashr1* and *ashh2* mutants. Furthermore, *PR1* gene expression was highest in the *ashr3* mutant, while H3K4me2 levels at the *PR1* promoter region are reduced in both the *ashr1* and *ashh2* mutants upon infection by DC3000 (De-La-Pena et al., 2012). This result demonstrates that the ASH1 group H3K4 methyltransferases have both overlapping and distinct roles in the plant defense against pathogens.

Collectively, the active H3K4 and H3K36 methylation states, which are catalyzed by SET domain protein, have been implicated in the SA- and JA-mediated plant defense in *Arabidopsis*. These markers act as permissive marks for the basal expression of the defense genes or establishing the chromatin status for prompt induction when plants are challenged. In contrast, the removal of the repressive histone H3K27me3 state by the JmjC protein JMJ705 in rice also plays important roles in defense-related gene expression. When induced by a stress signal or pathogen infection, JMJ705 is involved in the methyl jasmonate-induced removal of H3K27me3 and preferential biotic stress-responsive gene activation, supporting the hypothesis that H3K27me3 maintains the resting state of defense genes under normal conditions (Li et al., 2013). FLD, a homolog of the human LSD1, was originally discovered to promote flowering time by negatively regulating the expression of flower repressor FLC (He et al., 2003; Liu et al., 2007). A forward genetic screen revealed that *Arabidopsis* requires FLD in order to respond to the SAR signals leading to the systemic accumulation of SA; the screen also revealed that FLD influences histone modifications at the promoters of *WRKY29* and *WRKY6* and thereby enables a robust activation of SA signaling in response to subsequent exposure to virulent pathogens (Singh et al., 2013, 2014b).

### Histone Mono-Ubiquitination

In *Arabidopsis*, histone H2B mono-ubiquitination is catalyzed by the RING E3 ligases histone mono-ubqutinatio1 (HUB1) and HUB2, which participate in various developmental process such as the control of flowering time, the cell cycle, seed dormancy, and circadian clock (Xu et al., 2009; Lolas et al., 2010; Bourbousse et al., 2012). Additionally, HUB1 is a regulatory component of plant defense against necrotrophic fungal pathogens. *Arabidopsis* plants with mutations in the HUB1 alleles are extremely susceptible to the necrotrophic fungi *B. cinerea* and *A. brassicicola*. Consistent with the plant cell wall functioning in resistance to necrotrophic fungi by acting as a physical barrier, the thickness of epidermal cell walls is reduced in the *hub1* mutant. This suggests that HUB1 may enhance defense by increasing the thickness or otherwise modifying epidermal cell walls. Interestingly, HUB1 interacts with MED21, a subunit of the Mediator complex, in regulating the function of RNA polymerase II. *Arabidopsis* MED21 couples critical roles in disease resistance and embryo development based on the disease susceptibility and embryo-lethal phenotypes of plant lines with reduced MED21 gene expression. Thus, MED21 together with HUB1 controls critical components involved in the regulation of defense against necrotrophic fungal pathogens, suggesting a transcriptional role of Hub1-mediated histone mono-ubiquitination in defense (Dhawan et al., 2009). In contrast, responses to the bacterial pathogen *P. syringae* are unaltered in *hub1* plants. However, a recent report showed that both Hub1 and Hub2 regulate the expression of the R genes *SNC1* and *Rpp4* (Zou et al., 2014). In the auto-immunity mutant *bon1*, which is a negative regulator of the NB-LRR-encoding R gene *SNC1* and other R-like genes (Yang and Hua, 2004; Li et al., 2007), loss of function in *HUB1* or *HUB2* reduces *SNC1* up-regulation and suppresses the *bon1* auto-immune phenotypes. Thus, HUB1 and HUB2 mediate histone 2B (H2B) mono-ubiquitination directly at the *SNC1* R gene locus to regulate its expression. This is another example of how the immune response can be fine-tuned by histone modifications at an R gene locus (Zou et al., 2014).

### Chromatin Remodeling

In addition to being affected by covalent histone modifications, plant defense can also be affected by chromatin-remodeling factors that regulate R gene function and specific JA or SA pathways. The *Arabidopsis* genome encodes more than 40 ATP-dependent chromatin-remodeling factors, which can be subdivided into at least five families based on their ATPase subunits. In the broad SWI2/SNF2 protein family, DDM1 functions antagonistically to MOS1 in regulating the expression of the R gene *SNC1* (Li et al., 2010). SWR1, a component of the *Arabidopsis* SWR1-like complex that replaces the histone H2A with the histone variant H2A.Z, is required for maintaining the repression of SA-dependent defense genes in unstressed plants (March-Diaz et al., 2008). SWI/SNF class chromatin remodeling ATPase SPLAYED (SYD) can be directly recruited to the promoters of selected genes, i.e., *PDF1.2a, VSP2*, and *MYC2*, downstream of the JA and ET signaling pathways. Therefore, SYD is required for the expression of these genes and for resistance against the necrotrophic pathogen *B. cinerea* but is not required for resistance against *P. syringae* (Walley et al., 2008).

## Concluding Remarks and Perspectives

**FIGURE 1:**
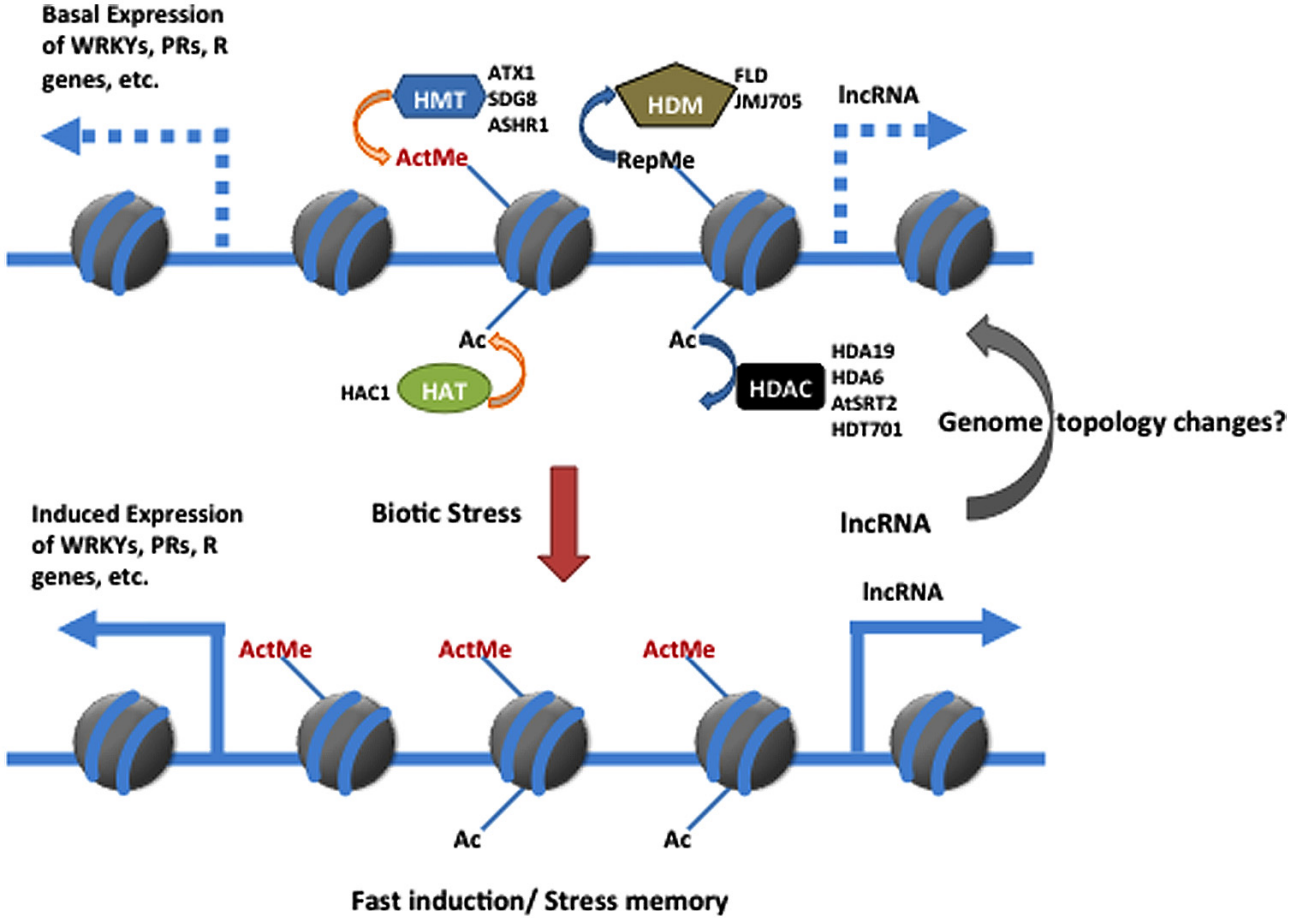
**Simplified model for participation of chromatin modification in regulating plant immunity against biotic stress.** Histone modification changes in defense-related gene can be achieved through methylation/demethylation and/or acetylation/deacetylation by antagonistic interaction between HMT and HDM or HAT and HDAC Each enzymes catalyzed different modification in regarding its roles in plant immunity is described in literature. The hypothetical involvement of the IncRNA in regulating the dynamin defense gene expression through the modulation of chromatin architecture is proposed as well. ActMe, active methylation marker; RepMe, Repressive methylation marker; HMT, histone methyltransferase; HDM, histone demethylase; HAC, histone acetylase; HDAC, histone de acetylase; IncRNA, long non-coding RNA; PR, pathogenesis-related; R, Resistance.

Recent research has increased our understanding of how chromatin modifications and remodeling affect defense in the model plants *Arabidopsis* and rice. Based on current evidence and as summarized in **Figure 1**, histone modifications in plant defense responses can be grouped as follows: (1) active histone marks that establish a basal expression level of the defense genes to enable an effective induction when the plant is challenged; (2) repressive histone modifications that prevent unnecessary activation of defense-related genes under normal growth conditions; (3) histone modifications that are induced after pathogen infection and that induce or reinforce the expression of defense-related genes; and (4) histone/chromatin changes that occur in response to biotic or abiotic stresses and that can be transmitted to the next generation. In the future, a combination of new genomic and proteomic approaches should be used to identify the targets of the epigenetic-related enzymes and other factors that are involved in the regulation of plant immunity. In addition, only a few histone-modifying enzymes have been investigated. Large-scale screens and characterization of epigenetic mutants should help increase our understanding of the histone-modifying enzymes involved in the chromatin changes that occur when plants defend against pathogens. Moreover, three-dimensional structure plasticity of genomes establishes fine-tune feature in gene expression modulation rather than defined by its linear context. Emerging evidence showed that lncRNAs (long non-coding RNAs) and chromatin remodeling complexes are shaping the dynamic genome topology through chromatin loops to regulate dynamic gene expression in response to the environmental cues (Ariel et al., 2014; Jegu et al., 2014). Considering that the global genome structure is impacted in many diseases in animal systems and the participation of lncRNAs in nuclear architecture, the association between non-coding RNAs and the genome topology related to chromatin marks and organization remains an unexplored area in plant immunity.

## Author Contributions

BD and G-LW wrote the manuscript.

## Conflict of Interest Statement

The authors declare that the research was conducted in the absence of any commercial or financial relationships that could be construed as a potential conflict of interest.
